# Supplementary material for: Experimental study on the coupling control of rock blasting vibration and rock fragmentation size
Source: PLoS One. 2026 Apr 1;21(4):e0338396. doi: 10.1371/journal.pone.0338396 (PMC13043048; doi:10.1371/journal.pone.0338396)
Supplement: Attachment — Submitted filename: Review comments.doc [file pone.0338396.s002.doc]

（1）The title of this manuscript is "Experimental study on the coupling control of rock blasting vibration and crushing fragmentation size", but why does the first paragraph of the Introduction only mention blasting vibration and not the crushing fragmentation size?

（2）The references in the first paragraph of the Introduction of this manuscript start from [4], not [1]. In addition, the text "Error! Reference source not found" appears multiple times in the Introduction.

（3）Fig. 6 was not cited in the text, and the reviewer was unaware of its function.

（4）The formats of the references are inconsistent, such as [1] and [2].

There are too many similar format issues in the manuscript. It is suggested that the authors make revisions and resubmit it.
