# Supplementary material for: Experimental study on the coupling control of rock blasting vibration and rock fragmentation size
Source: PLoS One. 2026 Apr 1;21(4):e0338396. doi: 10.1371/journal.pone.0338396 (PMC13043048; doi:10.1371/journal.pone.0338396)
Supplement: Attachment — Submitted filename: Response to Reviewers.docx [file pone.0338396.s005.docx]

**Response to Reviewers**

**Part I** is the response to journal requirements. **Part II** is the response to reviewers' comments.

**Part I**: Response to Journal Requirements

**Question** 1. We note that the grant information you provided in the ‘Funding Information’ and ‘Financial Disclosure’ sections do not match. Please ensure that you provide the correct grant numbers for the awards you received for your study in the ‘Funding Information’ section.

**Response**: It was modified. The grant numbers in the ‘Funding Information’ section have been verified and unified with the official records, ensuring consistency with the financial disclosure. The correct grant numbers are: The general project of Hubei Provincial Key Laboratory of Blasting Engineering(BL2021-11),National Natural Science Foundation of China(52274136,51904210) and Natural Science Foundation of Hubei Province(2024AFB766).

**Question** 2. Please state what role the funders took in the study. If the funders had no role, please state the standard statement.

**Response**: It was modified. The role of funders has been added to the cover letter and ‘Funding Information’ section: "The funders had no role in study design, data collection and analysis, decision to publish, or preparation of the manuscript."

**Question** 3. Please upload a Response to Reviewers letter with a point-by-point response to each point made by the Editor and/or Reviewers.

**Response**: This letter serves as the point-by-point response to all comments from the Editor and reviewers, covering all raised issues with detailed modification explanations.

**Question** 4. You note that your data are available within the Supporting Information files, but no such files have been included. Please upload your minimal data set as a Supporting Information file or to a public repository.

**Response**: It was modified. The minimal data set has been uploaded as Supporting Information.

**Question** 5. Please complete an Authorship Change Request form for the updated author list and obtain confirmation from all authors.

**Response**: It was modified. The Authorship Change Request form has been completed, signed by all authors confirming their approval of the author list (Li He, Wuyi Zhang, Yongming Zhao, Sheng Peng, Yingkang Yao, Shasha Chen, Maolin Wang). The form is uploaded as a separate file labeled ‘Other’.

**Question** 6. Please validate your ORCiD iD in your Editorial Manager account.

**Response**: It was completed. The ORCiD iDs of all authors have been validated in the Editorial Manager account.

**Question** 7. Please ensure that all figures and tables are referenced in the text, and correct inconsistent figure/table numbering.

**Response**: It was modified.

**Question** 8. Please unify the format of references and supplement missing information (e.g., DOI).

**Response**: It was modified. All references have been standardized according to PLOS ONE’s format requirements (Author. Title[J]. Journal Name, Year, Volume(Issue): Pages. DOI).

**Part II**: Response to Reviewers' Comments

**Response to Reviewer 1**

Many thanks for the reviewer's valuable comments. We have carefully revised the manuscript in terms of content, logic, and format. The modified content is marked in red in the revised manuscript.

**Comment** 1. Limited Realism and Representativeness of Laboratory Experiments. The manuscript should explicitly discuss the discrepancy between concrete blocks and real rock masses, and articulate how laboratory results can be extrapolated to realistic conditions.

**Response**: It was modified. We have clarified the three core differences between the 20×20×20 cm homogeneous C50 concrete specimens and the in-situ jointed gneiss rock masses (the target rock mass in field tests) in the 2. Test Progress section, combined with the mechanical parameter comparison in Table 1:first, the experimental specimens are homogeneous concrete materials without natural joints; second, the size of the experimental specimens is limited by the laboratory scale; third, the laboratory provides a standard and controllable blasting environment and signal acquisition environment.

We have supplemented the extrapolation logic of laboratory results to practical engineering scenarios in the 2. Test Progress and 6. Field Test Validation sections, based on similarity principle compliance and core mechanism universality

**Comment** 2. Experimental Setup Description Is Incomplete. No actual photographs of the laboratory test arrangement are provided.

**Response**: It was modified.

**Comment** 3. Use of Electronic Detonators Inside Small Holes. The fragmentation bias caused by metal-shell electronic detonators is not acknowledged.

**Response**: This study focuses primarily on the effects of delay time on blasting vibration and fragmentation size in concrete blasting experiments. While the fragmentation effect induced by the detonator casing itself may indeed impact the crushing performance of small-scale concrete specimens, this influence does not interfere with the selection of the optimal delay time combination.

**Comment** 4. Practical Relevance of the Recommended Delay Times (3–4 ms). The authors must explain the correlation between laboratory micro-delays and field-used delays.

**Response**: It was modified. The delay time interval of actual project can be calculated based on the geometric similarity ratio of laboratory parameters and field engineering paramet When applied to actual engineering, the delay time can be scaled according to the hole spacing. The field test in this study also verified that the optimal delay combination (38ms/68ms) scaled from the laboratory interval achieves excellent vibration reduction effects, confirming the practical relevance of the laboratory-derived micro-delays.The "Conclusions" section has been supplemented with relevant explanations

**Comment** 5. Reliability of Fragmentation-Related Conclusions. The fragmentation conclusions are overinterpreted given the experimental limitations.

**Response**: It was modified. The "Conclusions" section has been revised to moderate the interpretation of fragmentation results: "The laboratory results show that the optimal delay scheme (3ms/3ms) reduces the average fragment size and large fragment size by 10% and 9.3%, respectively. This conclusion is applicable to small-scale blasting of homogeneous brittle materials. For rock masses with developed joints, the fragmentation effect should be further verified by field tests, and the delay interval can be adjusted according to the structural characteristics of the rock mass."

**Comment** 6. Incorrect Attribution and Referencing of the Swebrec Function. The function was developed by Finn Ouchterlony, not "Swebrec et al.".

**Response**: It was modified.

**Response to Reviewer 2**

Many thanks for the reviewer's careful comments. We have thoroughly revised the manuscript to address the format and content issues. The modified content is marked in red in the revised manuscript.

**Comment** 1. The first paragraph of the Introduction only mentions blasting vibration and not crushing fragmentation size, which is inconsistent with the title.

**Response**: It was modified.

**Comment** 2. The references in the first paragraph of the Introduction start from [4], not [1], and "Error! Reference source not found" appears multiple times.

**Response**: It was modified.

**Comment** 3. Fig. 6 was not cited in the text, and its function is unclear.

**Response**: It was modified.

**Comment** 4. The formats of the references are inconsistent (e.g., [1] and [2]).

**Response**: It was modified.

**Response** to Reviewer 3

Many thanks for the reviewer's insightful comments. We have revised the manuscript to improve logic, completeness, and scientific rigor. The modified content is marked in red in the revised manuscript.

**Comment** 1. The abstract only mentions the "interval-based delay optimization framework" but fails to briefly explain its core logic (e.g., how to achieve coupled control through intervals rather than fixed values). It is recommended to add 1-2 sentences about the core principle of the framework to enhance the completeness of the abstract..

**Response**: It was modified. The abstract has been supplemented with the core logic of the framework: “Unlike traditional fixed-value delay designs, this framework responds to the variability of blasting parameters and rock mass properties by determining a reasonable delay time interval (instead of a single fixed value). It integrates Hanukayev's theory for delay time calculation, the Swebrec function for fragmentation evaluation, and the 'vibration reduction rate' for vibration assessment, realizing synergistic control of vibration mitigation and fragmentation optimization.”

**Comment** 2. Although the introduction reviews domestic and foreign studies, it does not clearly point out specific literature limitation cases of the current "single-factor focused research" (e.g., a scholar's study only focuses on vibration without involving fragmentation). It is recommended to supplement the limitation analysis of 1-2 typical literatures to strengthen the persuasiveness of the research gap..

**Response**: It was modified.

**Comment** 3. In the experimental design of Section 2.1, it is mentioned that "vibration measurement points 5, 6, and 7 are arranged on the lateral side of the free face," but only the reverse and forward direction data of the free face are analyzed in Sections 4.1 and 4.2, with no mention of the processing results of the lateral data. It is recommended to supplement the analysis of lateral PPV changes, vibration attenuation rate, and fragment distribution to achieve the unity of experimental design and data processing/analysis.

**Response**: It was modified.The experimental protocol has been adjusted. Given the small specimen size (200 mm×200 mm×200 mm), mutual interference was likely to occur between the lateral sensors and the free surface, resulting in unreliable data from these sensors. Thus, the arrangement of lateral sensors (i.e., those oriented perpendicular to the free surface) has been removed in the revised experimental design.

**Comment** 4. When deriving the delay time based on Hanukayev's theory in Section 3.1, only formulas are provided without substituting experimental parameters (such as the longitudinal wave velocity C and minimum resistance line W of C50 concrete) to demonstrate the calculation process. It is recommended to add an example of substituting key parameters for calculation to make the derivation process more traceable.

**Response**: It was modified. The mechanical parameters of C50 concrete and jointed gneiss have been supplemented, as shown in Table 1.

**Comment** 5. Section 4.2 mentions "performing Fast Fourier Transform (FFT) on blast vibration data to derive the dominant frequency," but the frequency domain analysis method (such as FFT parameter settings and frequency resolution calculation) is not separately clarified in "3 Theoretical Derivation" or "4 Result Analysis." It is recommended to add a new subsection or supplementary paragraph titled "Frequency Domain Analysis Method" to improve the analysis logic.

**Response**: It was modified. “Frequency Domain Analysis Method” has been added:Blasting vibration signals contain both time-domain and frequency-domain information. The time-domain index (PPV) was used to evaluate the vibration intensity, while the frequency-domain characteristics (dominant frequency) were extracted via Fast Fourier Transform (FFT) to assess the resonance risk.the original time-domain vibration data were processed using MATLAB R2023a with the following key settings: a sampling frequency of 10 kHz, a 5% Hanning window to reduce spectral leakage, a data length of 1024 points (frequency resolution = 9.77 Hz), and the dominant frequency was determined as the frequency with the maximum amplitude in the resulting power spectrum.

**Comment** 6. The experiment uses C50 concrete instead of natural rock. Only the mix ratio and strength grade of the concrete are mentioned in Section 2.1, without explaining the basis for the similarity of its mechanical parameters (such as elastic modulus, Poisson's ratio, and compressive strength) to the target natural rock mass (e.g., limestone and granite commonly found in open-pit mines). It is recommended to add a comparison table of mechanical parameters between concrete and natural rock mass (literature data can be cited) to verify the rationality of the model.

**Response**: It was modified. A new table (Table 1) has been added as Supporting Information, comparing the mechanical parameters of C50 concrete and natural rock masses

**Comment** 7. Some figures (such as Figure 1, Figures 2-5) have low resolution and missing key unit labels. It is recommended to increase the resolution of all figures to 300 dpi, correct image display issues, and unify the units of figures and tables.

**Response**: It was modified.

**Comment** 8. The "optimized interval of delay time (tk=3–4 ms, tp=1–3 ms)" is proposed in Section 5.3, but the advantages of the interval-based delay time determination method compared with the traditional fixed-value method are not reflected. It is recommended to supplement the explanation of the advantages to enhance the expression of innovation.

**Response**: It was modified. The "Conclusions" section has been supplemented with advantages explanation: “Compared with traditional fixed-value delay design, the interval-based method has three core advantages:first, facilitates flexible selection of delay time within the interval by constructors based on actual conditions.second, ensures safe superimposed vibration intensity while avoiding poor rock fragmentation from excessive delays.third, adapts to changes in blast center distances and geological conditions, enhancing the applicability of the delay scheme.”

**Comment** 9. In Table 3 "Fragmentation Size Table for Inter-hole Delay" in Section 4.1, the Xmax (19.8 cm) of Group 3 is much higher than that of other groups, but the reason for the increased fragments caused by the delay time (tk=2 ms) of this group (such as insufficient superposition of stress waves and inadequate crack propagation) is not analyzed. It is recommended to add 1-2 sentences of mechanism analysis on abnormal data to improve the depth of result interpretation..

**Response**: It was modified. The "Inter-hole delay time data analysis" section has been supplemented with detailed mechanism analysis: “Group 3 (tk=2ms) has the highest Xmax (19.8cm) because the short delay time leads to insufficient superposition of stress waves between adjacent holes. The stress wave from the first detonated hole has not fully propagated to the adjacent hole when the second hole detonates, resulting in inadequate crack expansion and incomplete fragmentation of the concrete specimen.”

**Comment** 10. When using the Swebrec function to evaluate the fragment size in Section 3.2, only the formula is given without explaining the calculation process of the "fluctuation exponent b" (e.g., how to solve it with the least-squares method through sieving data). It is recommended to supplement the key steps of calculating the b value (such as sample size and goodness of fit R²) to enhance the repeatability of the method.

**Response**: It was modified. Plot the Swebrec fitting distribution diagram based on the experimental fragment size data following these steps:1. Collect and sieve fragment samples to obtain cumulative mass percentage P(x); 2. The parameter *b* is calculated by fitting the cumulative mass percentage data P(x) and the corresponding fragment size x using the least-squares method.; 3. Verify the fitting validity with the coefficient of determination *R²*≥0.95

**Comment** 11. There are ambiguous expressions and grammatical errors in some sentences of the paper. It is recommended to recheck the grammar to make the expression professional and accurate.

**Response**: It was modified.

**Comment** 12. The conclusion mentions that "PPV shows exponential attenuation in the free face direction and linear attenuation in the reverse direction," but it is not supported by specific data in the paper (such as the forward attenuation coefficient and reverse attenuation slope under a certain group of delays). It is recommended to supplement key data (e.g., the forward PPV of Group 4 decreases from X cm/s to Y cm/s, conforming to the exponential law y=ae^(-bx)) to enhance the credibility of the conclusion.

**Response**: It was modified. The "Conclusions" section has been supplemented with specific data: "Free face direction PPV of Group 4 (tk=3ms, tp=3ms) decreases exponentially from 10.01cm/s (0.4m blast center distance) to 2.14cm/s (1.7m blast center distance), conforming to the exponential law y=10.01e^-0.8x^ (R²=0.96). Free face reverse PPV decreases linearly from 14.06cm/s to 3.46cm/s, following the linear law y=-4.79x+13.82 (R²=0.95)."

**Comment** 13. The format of references is inconsistent. Some references (such as [21], [25]) lack complete DOIs or journal names. It is recommended to unify and standardize the format according to "Author. Title[Document type identifier]. Journal name, Year, Volume(Issue): Pages. DOI." and supplement missing DOIs and journal names.

**Response**: It was modified.

**Comment** 14. For the parameter settings of "inter-hole spacing of 100 mm and row spacing of 70 mm" in the experimental design, the basis for the similarity ratio between this size and the actual deep-hole blasting in open-pit mines (e.g., inter-hole spacing of 5-10 m) is not explained. It is recommended to supplement the design logic of the similarity ratio to illustrate the correlation between the model experiment and engineering practice.

**Response**: It was modified. The laboratory’s inter-hole (100 mm) and row spacing (70 mm) yield a 1.43:1 ratio, matching the 1.0–1.5:1 range for open-pit loose blasting. This ensures consistent stress wave interference between the model and field blasting. For on-site applications, delay times should be recalculated via Hanukayev's theory, based on the geometric similarity ratio of mine benches to lab specimens and field rock parameters.The “Test progress” section has been supplemented with relevant analysis: This study is applicable to small-scale blasting of homogeneous brittle materials (e.g., rock masses with Kv≥0.8 like roadway excavation), providing delay time trend references. For large open-pit deep-hole blasting, on-site parameter calibration is required. Despite differences, the test model follows a 1:20-1:50 geometric similarity ratio to actual blasting, with 1.07g TNT-equivalent charge and three soil-compacted sides simulating surrounding rock constraints.

**Comment** 15. When analyzing the dominant frequency law in Section 5.2, only "the dominant frequency attenuates with the increase of the blast center distance" is mentioned, without explaining the practical significance of the change in dominant frequency for "avoiding structural resonance" (e.g., the dominant frequency of a certain delay scheme is always higher than the resonance frequency of buildings). It is recommended to supplement the correlation analysis between the dominant frequency and engineering safety to strengthen the engineering value of the research.

**Response**: It was modified. The “Inter-row delay time data analysis” section has been supplemented with relevant analysis: “The main frequency of all optimized delay schemes (45-75Hz) is much higher than the resonance frequency of common ground structures (1-10Hz). For example, the optimal 3ms/3ms scheme achieves a main frequency of 58-72Hz, which avoids vibration amplification caused by resonance, effectively reducing the risk of structural damage in blasting operations near surrounding buildings.”

**Response to Reviewer 4**

Many thanks for the reviewer's constructive comments. We have made targeted revisions to improve the manuscript’s clarity and scientific contribution. The modified content is marked in red in the revised manuscript.

**Comment** 1. In the first paragraph of the introduction, references [1-3] are missing, and references [4-7] are cited improperly. Moreover, two citation errors also appear in the second paragraph of the introduction. This gives readers the impression that the research in this manuscript is not yet fully developed.

**Response**: It was modified.

**Comment** 2. Figures 2-7 are presented as separate charts, but they actually form a set of images. It is recommended to consolidate them into a multi-panel chart module to enhance clarity and compactness. Furthermore, regarding the interpretation of Figures 2-7, presenting the impact patterns with concrete data would make the research findings more persuasive. Regarding Figure 8-11, the same recommendation applies..

**Response**: It was modified.

**Comment** 3. The text in the images within the manuscript is not very clear and is difficult to read, requiring better design of the text within the images.

**Response**: It was modified.

**Comment** 4. Fig. 13 should provide references.

**Response**: It was modified.

**Comment** 5. The interval-based delay optimization framework proposed in this manuscript is not clearly defined, preventing readers from systematically learning the underlying theory and methodology. Although the manuscript presents some experimental findings, its scientific contributions remain somewhat unclear.

**Response**: It was modified. A new subsection “Interval-based Delay Optimization Framework” has been added to the “This study’s interval-based delay optimization framework follows four key steps: calculating initial inter-hole and inter-row delay times via Hanukayev's theory; fixing the initial inter-row delay time to screen the optimal inter-hole delay time through comprehensive vibration and fragmentation evaluation; fixing the optimal inter-hole delay time to screen the optimal delay combination via the same method; and adjusting the laboratory delay interval by geometric similarity ratio for field verification.”
