# Supplementary material for: Experimental study on the coupling control of rock blasting vibration and rock fragmentation size
Source: PLoS One. 2026 Apr 1;21(4):e0338396. doi: 10.1371/journal.pone.0338396 (PMC13043048; doi:10.1371/journal.pone.0338396)
Supplement: Attachment — Submitted filename: comments.docx [file pone.0338396.s004.docx]

In response to the issues raised by the reviewer previously, although the authors have made some revisions, there are still many formatting problems in the current manuscript. The reviewer have listed the more obvious problems as follows:

(1) Citation of references in the text: For multiple consecutive references, such as the first five references in the text, the citation format should be [1-5], rather than "[1][5]". Similarly, "[17]-[20]" in the manuscript is also not standard. Additionally, reference [7] in the Introduction is not cited.
(2) Generally, a space should be added between numbers and units throughout the text, but the authors have not consistently followed this rule, such as "0.5m" and "45-75 Hz".
(3) For the references listed at the end of the manuscript, the format is still not uniform (e.g., [1], [4]) and not standard (e.g., [5]). The reviewer have provided examples of both Chinese and English reference lists as follows:
[1] Yang Z., Tao M., Memon M.B., Zhuang D.D., Zhao Y. Microwave irradiation-induced deterioration of rock mechanical properties and implications for mechanized hard rock excavation [J]. Journal of Rock Mechanics and Geotechnical Engineering, 2024, 24: 00308.
[2] Li X.B., Zhou J., Wang S.F., Liu B. Review and practice of deep mining for solid mineral resources [J]. The Chinese Journal of Nonferrous Metals, 2017, 27(06): 1236-1262. (in Chinese)

Regarding the above-mentioned problems existing in the manuscript, the reviewer did not list them one by one. It is suggested that the authors carefully revise the entire text.
