# Supplementary material for: Experimental study on the coupling control of rock blasting vibration and rock fragmentation size
Source: PLoS One. 2026 Apr 1;21(4):e0338396. doi: 10.1371/journal.pone.0338396 (PMC13043048; doi:10.1371/journal.pone.0338396)
Supplement: Attachment — Submitted filename: Response_to_Reviewers_auresp_2.docx [file pone.0338396.s006.docx]

**Response to Reviewers**

Thank You Note

We sincerely appreciate the editorial team and all reviewers for their valuable comments and constructive suggestions, which have significantly helped improve the quality and scientific rigor of this manuscript. We have carefully addressed each comment and made corresponding Responses to the manuscript. All modifications are marked in red in the revised manuscript. Below is the point-by-point response to each comment.

---

**Response to Reviewer 2**

**Comment 1**: Citation of references in the text: For multiple consecutive references, such as the first five references in the text, the citation format should be [1-5], rather than "[1][5]". Similarly, "[17]-[20]" in the manuscript is also not standard. Additionally, reference [7] in the Introduction is not cited.

**Response**: It was modified.

1. Unified all consecutive reference citations to the "[start-end]" format: changed "[1][5]" to "[1-5]" and "[17]-[20]" to "[17-20]".

2. Supplemented the citation of Reference [7] in the Introduction: Added "For example, Zhou et al. studied the attenuation law of vibration frequency during the propagation of blasting seismic waves, providing a theoretical basis for frequency-domain control of blasting vibration[7]." after the sentence "To realize reasonable control of blasting vibration, researchers have used theoretical inference, numerical simulation, model construction, field tests, and other research methods to analyze the impact of different vibration reduction measures on blasting vibration[6-7]."

**Comment 2**: Generally, a space should be added between numbers and units throughout the text, but the authors have not consistently followed this rule, such as "0.5m" and "45-75 Hz".ts

**Response:** It was modified.Unified all numbers and units in the full text by adding a space (e.g., "0.5 m", "45–75 Hz"). Special composite units (e.g., "kg/m³", "m/s") retain no internal space, and only the number and unit are separated by a space. All instances (abstract, text, tables, figures) have been revised, such as "3ms" → "3 ms", "100mm" → "100 mm", "0.4m" → "0.4 m".

**Comment 3**: For the references listed at the end of the manuscript, the format is still not uniform (e.g., [1], [4]) and not standard (e.g., [5]). The reviewer have provided examples of both Chinese and English reference lists as follows:
[1]Yang Z., Tao M., Memon M.B., Zhuang D.D., Zhao Y. Microwave irradiation-induced deterioration of rock mechanical properties and implications for mechanized hard rock excavation [J]. Journal of Rock Mechanics and Geotechnical Engineering, 2024, 24: 00308.
[2]Li X.B., Zhou J., Wang S.F., Liu B. Review and practice of deep mining for solid mineral resources [J]. The Chinese Journal of Nonferrous Metals, 2017, 27(06): 1236-1262. (in Chinese)
Regarding the above-mentioned problems existing in the manuscript, the reviewer did not list them one by one. It is suggested that the authors carefully revise the entire text.

**Response:**  It was modified. We have revised and unified the format of most references in strict accordance with the required standards. However, some references (e.g., Reference [4]) are publicly available online on the journal’s website but have not yet been formally published in print, which results in the lack of formal page numbers and DOI in their format.We will update the information promptly once the formal publication details are released.

---

**Response to Reviewer 4**

Many thanks for the reviewer's constructive comments. We have made targeted revisions to improve the manuscript’s clarity and scientific contribution. The modified content is marked in red in the revised manuscript.

**Comment** 1. In the first paragraph of the introduction, references [1-3] are missing, and references [4-7] are cited improperly. Moreover, two citation errors also appear in the second paragraph of the introduction. This gives readers the impression that the research in this manuscript is not yet fully developed.

**Response**: It was modified. We rechecked and consolidated the original continuous citations [1-5] and [6-7] in the Introduction, verified the relevance of each literature to the corresponding content, supplemented brief connections between citations and discussions to avoid disconnectedness, corrected misnumbered references and logical inconsistencies in the second paragraph, and maintained the manuscript’s original citation style while ensuring accuracy and comprehensiveness.

**Comment** 2. Figures 2-7 are presented as separate charts, but they actually form a set of images. It is recommended to consolidate them into a multi-panel chart module to enhance clarity and compactness. Furthermore, regarding the interpretation of Figures 2-7, presenting the impact patterns with concrete data would make the research findings more persuasive. Regarding Figure 8-11, the same recommendation applies..

**Response**: It was modified.We consolidated Figures 2-11 into multi-panel chart modules (each containing 2-4 subplots) with consistent axes and legends for compactness, and supplemented specific experimental data (e.g., PPV values, vibration reduction rates, fragmentation size parameters, fitting coefficients) in figure interpretations to enhance the persuasiveness of research findings.

**Comment** 3. The text in the images within the manuscript is not very clear and is difficult to read, requiring better design of the text within the images.

**Response**: It was modified. We optimized the text design in all figures by increasing font size to 11 pt, adjusting color contrast for better visibility, unifying fonts and alignment, and eliminating redundant labels—ensuring all text is clearly legible in high-resolution (300 dpi) files.

**Comment** 4. Fig. 13 should provide references.

**Response**: It was modified. We supplemented a relevant classic literature citation for Fig. 13 (field test vibration attenuation curve), which has been standardized in the reference list and clearly marked in the figure caption to comply with academic norms.

**Comment** 5. The interval-based delay optimization framework proposed in this manuscript is not clearly defined, preventing readers from systematically learning the underlying theory and methodology. Although the manuscript presents some experimental findings, its scientific contributions remain somewhat unclear.

**Response**: It was modified. A new subsection “Interval-based Delay Optimization Framework” has been added to the “This study’s interval-based delay optimization framework follows four key steps: calculating initial inter-hole and inter-row delay times via Hanukayev's theory; fixing the initial inter-row delay time to screen the optimal inter-hole delay time through comprehensive vibration and fragmentation evaluation; fixing the optimal inter-hole delay time to screen the optimal delay combination via the same method; and adjusting the laboratory delay interval by geometric similarity ratio for field verification.”

---

**Response to Reviewer 5**

**Comment 1**: The similarity ratio of the experimental model is used as a reference, reducing the rationality of the article.

**Response:** It was modified. To enhance the rationality of the manuscript, we have removed the relevant description of the experimental model’s similarity ratio, including deleting the sentence “Despite differences, the test model follows a 1:20-1:50 geometric similarity ratio to actual blasting, with 1.07 g TNT-equivalent charge and three soil-compacted sides simulating surrounding rock constraints” to avoid potential ambiguity about the model’s reliability.

**Comment 2**: The reviewer has pointed out that the effect of the electronic detonator shell on the fragmentation effect is not sufficiently discussed. The author responded that it "does not affect the selection of the optimal delay combination." However, from the perspective of academic rigor, it is recommended to clearly state in the 'Limitations' section: the detonator shell in small-sized specimens may have an additional impact on fragmentation, and the differences in charge structures need to be considered during field applications.

**Response:**It was modified. To enhance academic rigor, we have added a “Discussion” paragraph front the “Conclusions” section as recommended, with the content as follows: This study has several limitations that need to be considered in engineering applications: (1) The digital electronic detonators used in the laboratory have a metal shell design, which may exert additional local impact on the fragmentation of small-sized concrete specimens (200 mm × 200 mm × 200 mm). Although this impact does not affect the screening of the optimal delay time combination, special attention should be paid to the difference in charge structure between the laboratory (using only detonators) and the field (using bulk explosives) when applying the research results to actual blasting projects. (2) The experimental specimens are homogeneous C50 concrete without natural joints, which is essentially different from the jointed rock mass structure in the field. Therefore, for large-scale open-pit blasting projects, on-site parameter calibration must be performed." The added content is marked in red in the revised manuscript.

**Comment 3**: The text mentions using the least squares method to fit parameter b, but the b values and goodness of fit R² for each group are not provided. It is recommended to supplement the b values and R² in Tables 4 and 5 to demonstrate the reliability of the fitting.

**Response:** It was modified.We have supplemented the shape parameter *b* and goodness-of-fit coefficient R^2^ for each group into Tables 4 and 5 as recommended. The *b* values are calculated based on the *X_80_* fragmentation characteristic points via the least squares method, which exhibit high precision and can effectively reflect the large-fragment properties of concrete specimens during blasting. All groups show *R^2^* values close to 1 (≥0.978), indicating excellent fitting reliability and the rationality of the parameter selection, which further enhances the persuasiveness of the research findings.

**Comment 4**: It is recommended to try to understand the issues raised by the reviewers and refine the details of the article. Latest research work related with this topic can be referred in the introduction. Probabilistic stability analyses of two-layer undrained slopes. Fracture evolution and mechanical deterioration of granite under cyclic thermal and liquid nitrogen cryogenic impact.

**Response:**It was modified. Supplemented citations of the latest related studies in the "Introduction" section : Zhu et al. further verified the dominant effect of geotechnical material spatial variability on slope failure risk via probabilistic stability analysis of two-layer undrained slopes, which highlights the engineering necessity of precise blasting vibration control in open-pit projects [8].Li et al. investigated the fracture evolution and mechanical deterioration of granite under cyclic thermal and liquid nitrogen cryogenic impact, revealing the coupled damage mechanisms of rock mass fracture development and mechanical property degradation, which provides important experimental insights for understanding the rock dynamic fragmentation mechanism in blasting engineering[22].

**Comment 5**: The text points out that the optimal delay is a range (tk=3-4 ms, tp=1-3 ms) but does not explain how the boundaries of this range are determined, such as whether it is based on empirical summaries of experimental results or some theoretical threshold. It is suggested to add a theoretical explanation of the range boundaries in the 'Discussion' section or propose a general principle for determining the range.

**Response:** It was modified. To clarify the scientific basis for defining the optimal delay time range boundaries, we have revised and integrated the theoretical derivation and experimental verification basis into the first item of the Conclusions section. The revised content is as follows: (1)The study derived the theoretical optimal delay time value via the Hanukayev formula, and then determined the optimal delay time range (tk=3-4 ms, tp=1-3 ms) through comparative analysis of experimental PPV and blasting fragmentation data. The selection standard for this range is to compare all delay groups in parallel, identify the groups with the best and second-best vibration reduction and blasting effects respectively, and finally select the groups meeting both standards to delimit the optimal range. Specifically, the 3-4 ms inter-hole delay range corresponds to the interval with significantly lower PPV and higher fragmentation uniformity than other schemes, achieving a 54-70% reduction in PPV while maintaining 92% fragmentation uniformity.

---

All Responses have been completed, and the revised manuscript has been carefully checked for format consistency and logical coherence. We sincerely thank the editorial team and reviewers for their valuable time and efforts. We hope the revised manuscript meets the publication requirements of PLOS ONE.

Sincerely,

Wuyi Zhang

Corresponding Author

Faculty of Science

Wuhan University of Science and Technology

Wuhan 430065, Hubei, China

Tel: 18972228852

Email: 18972228852@163.com
